# Macrophage modulation accounts for the anti-inflammatory effect of *Hypnea cervicornis* agglutinin in rat arthritis induced by zymosan

**DOI:** 10.1007/s10719-026-10221-5

**Published:** 2026-06-22

**Authors:** Francisco Glerison da Silva Nascimento, Pedro Henrique de Souza Ferreira Bringel, Diego Freitas de Araujo, Mário Rogério Lima Mota, Maria Gleiciane de Queiroz Martins, Mayara Torquato Lima da Silva, Clareane Avelino Simplício Nobre, Benildo Sousa Cavada, Kyria Santiago do Nascimento, Rondinelle Ribeiro Castro, Ana Maria Sampaio Assreuy

**Affiliations:** 1 Laboratório de Fisio-Farmacologia da Inflamação (LAFFIN), Instituto Superior de Ciências Biomédicas, Universidade Estadual do Ceará, Av. Dr. Silas Munguba 1700, Fortaleza, CE 60714-903 Brazil; 2 Laboratório de Moléculas Biologicamente Ativas (BioMol-Lab), Departamento de Bioquímica e Biologia Molecular, Universidade Federal do Ceará, Campus do Pici, s/n; Bloco 907, Fortaleza, CE 60455-970 Brazil

**Keywords:** Cytokine, HCA, Joint inflammation, Algae lectin

## Abstract

Previous study demonstrated that *Hypnea cervicornis* lectin (HCA) reduces inflammation and nociception via interaction with the lectin carbohydrate-binding site, modulating gene expression of the interleukins IL-1β, TNF-α and of iNOS. This study evaluated the effect of HCA in the rat model of arthritis induced by zymosan in the tibiotarsal joint and the involvement of macrophage-derived mediators. In vitro, macrophages were stimulated with zymosan before being incubated with HCA and the supernatant was injected into the joint. In vivo, HCA was administered by intravenous route after intra-articular injection of zymosan, fMLP, or macrophages supernatant. Hypernociception, edema and leukocyte influx were evaluated in the joints, and inflammatory mediators in the macrophage supernatant or periarticular tissue. In vitro, HCA (100 µg/ml) reduced NO_2_^−^ and IL-1β in the supernatant of macrophages. In vivo, HCA (3 mg/kg) reduced articular hypernociception, edema and leukocyte influx elicited by zymosan, fMLP or by the supernatant of zymosan activated macrophages, as well as the zymosan-induced rolling and adhesion, and gene expression of iNOS and IL-1. HCA attenuated edema and leukocyte influx in the periarticular tissue, revealing preserved chondrocytes in the cartilage. In conclusion, HCA exerts anti-inflammatory effect in the arthritis induced by zymosan in rat tibio-tarsal joints, involving NO and IL-1 released by resident macrophages.

**Supplementary Information:**

The online version contains supplementary material available at 10.1007/s10719-026-10221-5.

## Introduction

Rheumatoid arthritis (RA) is a chronic and autoimmune articular inflammatory disease of unknown cause, affecting 0.5–1% of the population with predominance in women and higher incidence in the age group of 30–50 years [1].

The main features of RA include typical persistent synovitis, rheumatoid pannus, joint pain, and its progression involves the release of inflammatory mediators by resident cells [1, 2]. Activated macrophages produce and release mediators, including nitric oxide (NO) and inflammatory cytokines such as interleukin-1 (IL-1) and TNF-α, which play essential role in the development of inflammatory diseases [3, 4]. IL-1 is deeply involved in the pathogenesis of arthritis, being elevated in the synovial fluid of patients due to its release by macrophages and chondrocytes [5, 6]. The NO generated via activation of the inducible nitric oxide synthase (iNOS) is also one of the important mediators in RA [7].

The literature had reported the modulator role of endogenous lectins in RA [8]. Besides, exogenous lectins isolated from leguminous plants and algae are also shown to negatively modulate arthritis in human and rodents via recognition and binding to glycan structures present in the membranes of inflammatory cells [9, 10]. The lectin of *Canavalia ensiformis* (ConA) stimulates the macrophage migration inhibitory factor (MIF) in synovial fibroblasts [11], and that of *Maackia amurensis* seed ameliorates cartilage destruction in TNF-α-induced arthritis in mice [12]. In addition, a lectin from the green alga *Caulerpa cupressoides* reduces mechanical hypernociception and inflammation in rat zymosan-induced arthritis, involving mechanisms dependent on the inhibition of TNF-α, IL-1β, COX-2 and adhesion molecules [13, 14]. It is also known that lectins promote neutropenia in some clinical conditions [15]. However, this effect has not been elucidated yet in experimental models of arthritis.

*Hypnea cervicornis* is a red alga found along the Pacific coast of Japan and the Atlantic coast of Brazil [16]. The isolated lectin from *H. cervicornis* (HCA), also referred as agglutinin, is a polypeptide containing 90 amino acid residues (9196.6 Da) with binding specificity to glycoproteins of the mucin type [17]. Pharmacological studies using acute inflammation models have shown that HCA presents low acute toxicity, inhibits neutrophil migration and paw edema induced by carrageenan [17], and the hypernociception induced by prostaglandin E_2_ (PGE_2_) triggered by a mechanism involving NO [18]. In addition, previous study demonstrated that the intravenous (i.v.) post-treatment (2 h after induction), but not the pre-treatment with HCA, reduces inflammation and nociception in the rat model of tibio-tarsal arthritis induced by zymosan, via interaction with the lectin carbohydrate-binding site, and inhibition of the gene expression of the interleukin TNF-α and iNOS [10]. However, the participation of leukocyte mediators in the anti-inflammatory effect of HCA is not fully understood.

Considering that lectins interact with macrophage toll-like receptors (TLRs), modulating the synthesis of inflammatory mediators, such as NO and TNF-α [19], the aim of this study was to evaluate the effect of HCA in the rat model of arthritis induced by zymosan, along with the involvement of neutrophils and macrophage-derived mediators.

## Materials and methods

### Drugs and reagents

Zymosan, RPMI 1640, formyl-methionyl-leucyl-phenylalanine (fMLP) and ELISA kit for IL-1β were purchased from Sigma-Aldrich (Saint Louis - Missouri, USA); Brazol kit from LGC Biotecnologia (Cotia - São Paulo, Brazil); DNAse and reverse transcriptase SuperScriptTM IV First-Strand Synthesis System from Invitrogen^®^ (Carlsbad - California, USA). GelRed from Biotium (Fremont - California, USA); primers Oligo (dT) and dNTP from Thermo Fisher (Waltham - Massachusetts, USA); primers for β-actin, IL-1 and iNOS from Integrated DNA Technology-IDT (Coralville - Iowa, USA). All other chemicals were of analytical grade.

### Alga collection and lectin isolation

The red alga *Hypnea cervicornis* J. Agardh (Florideophyceae) was collected at the Pacheco Beach, Ceara State, Brazil (3.686830° S; 38.639854° W), frozen at −20 °C, converted into fine powder in liquid nitrogen, and the crude extract obtained by protein precipitation using ammonium sulphate. The *H. cervicornis* lectin (HCA) was purified by ion exchange chromatography and its purity and apparent molecular weight evaluated by 15% SDS-PAGE [20].

### Animals

Female Wistar rats (200–220 g) were maintained under controlled conditions (22–25 °C, 12 h light/dark cycle), receiving water and food *ad libitum*. The experimental protocols were conducted in accordance with NIH guidelines (publication No 85 − 23, revised 2011) and National Council of Control in Animal Experimentation (CONCEA), being approved by the Ethics Committee of the State University of Ceara Brazil (CEUA/UECE No 3207571/2014).

### Macrophage isolation and incubation with HCA

Rats received 3% thioglycolate (25 µl) via intra-articular (i.art.) and four days later, leukocytes were harvested by i.art. injection of 100 µl heparinized 1640 RPMI medium [21]. Macrophage monolayers were prepared by addition of 1 ml/well (10^6^ macrophages) into 24-well culture plates. Macrophage adhesion was allowed to proceed for 24 h at 37 °C in 5% CO_2_ atmosphere, and the non-adhered macrophages were removed by 3 successive washes with RPMI.

The experimental protocol was performed in triplicate using three experimental groups: RPMI (negative control); zymosan (positive control); and HCA. Plate-adhered macrophages were stimulated with zymosan (10 µg/ml) and 1 h later HCA (100 µg/ml) was added into wells, being incubated by 1 additional hour.

Supernatants were collected, centrifuged (3 g; 5 min) for dosage of IL-1 and nitrite by ELISA. Moreover, the supernatant was re-injected (25 µl) into rat tibio-tarsal joints for evaluation of ankle diameter, leukocyte migration and hypernociception.

### Zymosan-induced arthritis and lectin treatment

Most experimental protocols were performed using five experimental groups (*n* = 8 per group): sterile saline (negative control); zymosan (positive control); and HCA at three doses. Arthritis was induced by single intra-articular injection of zymosan (500 µg/25 µl) into the right tibio-tarsal joints under sedation (xylazine 10 mg/kg + ketamine 80 mg/kg, i.m.) [10]. Sham animals received 0.9% NaCl (sterile saline; 25 µl i. art.). HCA (0.1–3 mg/kg) or saline was administered (i.v.) 5.5 h after zymosan. Thirty minutes later, animals were evaluated for hypernociception, leukocyte migration, gene expression of IL-1 and iNOS.

An additional group of non-HCA treated animals was evaluated at 5,5 h after arthritis induction for the zymosan effect on hypernociception and leukocyte migration.

### HCA effect on neutrophil migration

To evaluate the per se effect of HCA, another group of non-arthritic rats received HCA (3 mg/kg; i.v.), and the leukogram was analyzed 30 min after injection. Furthermore, the tibio-tarsal joints were also stimulated with the neutrophil chemotactic agent fMLP (10 nM; i.art) 3.5 h before HCA (3 mg/kg; i.v.) and leukocyte migration evaluated 30 min later.

### Hypernociception and ankle diameter measurement

Animals were individually placed into plexiglas boxes with malleable mesh net floor to allow access to the ventral surface of hind paws, in which 6 consecutives mechanical pressure were applied using a polypropylene tip (4.15 mm^2^) coupled to digital algesimeter to cause joint flection [10]. Reduced mechanical threshold required to evoke the paw withdrawal response (g) is indicative of hypernociception. The rat’s ankle diameters (measured three times at each evaluation time) were measured using a digital caliper in the neutral position of the ankle [22].

### Leukocyte migration to synovial cavity

Tibio-tarsal joints were washed twice with 100 µl phosphate buffered saline (PBS), containing 10 mM disodium ethylenediamine tetra acetic acid (EDTA), and the intra-articular fluid was harvested. The total leukocyte count was performed in Neubauer chamber. For differential leukocyte count, the fluid was centrifuged (670 g) and the pellet resuspended in 50 µl PBS for preparation of smear slides stained with H&E [10].

### Leukocyte rolling and adhesion

Zymosan was injected (1 mg/100 µl) by intraperitoneal route and HCA (3 mg/kg; i.v.) was administrated 30 min before evaluation. Sham-animals received saline (100 µl; i.p.).

After HCA treatment (30 min) the animals were anesthetized, as previously mentioned, for evaluation of the steps of leukocyte migration. Median laparotomy was performed, and the mesenteric bed was spread over a siliconized glass plate, maintained at 37° C, and visualized by intravital microscope. The number of leukocyte rolling (cells moving over the endothelium with slower rate in relation to red cells) and leukocyte adhesion (no visible movement for 30 s) was recorded in 200 μm venules for 10 min [23].

### Gene expression for IL-1 and inducible NO synthase (iNOS)

Gene sequences from *Rattus norvegicus* were obtained from GeneBank database. Specific genes primer for iNOS, IL-1 and reference gene primer for β-actin were obtained using Primer3 (http://primer3.ut.ee). Forward (F) and reverse (R) primers were F-5’AAT GGC AAC ATC AGG TCG GC-3’and R-5’CGT ACC GGA TGA GCT GTG AAT − 3’ for iNOS; F-5’TCA TCT TTG AAG AAG AAG AGC CCG − 3’ and R-5’TCA GAC AGC ACG AGG CAT TT-3’ for IL-1; and F-5’GCA CCA CAC CTT CTA CAA TGA G-3’ and R-5’GGT CTC AAA CAT GAT CTG GGT C-3’ for β-actin. All primers were synthesized by IDT with 25 nt lenght. IL-1 and iNOS primers were used for quantitative evaluation for gene expression by quantitative real-time polymerase chain reaction (qRT-PCR).

Samples of rat joint tissues (100 mg) were macerated in liquid nitrogen, and total RNA was extracted using Brazol kit and treated with DNase. Total RNA was resuspended in RNase-free water and their concentration and quality determined by spectrophotometry (NanoVue) at 260 nm. RNA purity was checked at optical density ratio (OD260/OD280) between 1.8 and 2.0. RNA integrity was analyzed in 1.2% agarose gel staining with GelRed. For cDNA synthesis, 1000 ng of total RNA was incubated with 1 µl of reverse transcriptase MMLV (GE Healthcare), 1 µl of primer Oligo (dT) (Thermo Fisher) and 1 µl of dNTP (Thermo Fisher). Reverse transcription reactions (20 µl) were performed in thermoblock (42 °C; 15 min). The enzyme denaturation was performed by heating (95 °C; 2 min), followed by rapid cooling at 4 °C. RT-negative control sample was performed at the same conditions but without addition of total RNA. The cDNA concentration was determined by spectrophotometry at 260 nm.

qRT-PCR was performed using termocycler Bioer LineGene 9660 (Bioer, China) and software PCR LineGene 9660. GoTaq^®^ qPCR Master Mix kit (Promega) was used to amplify the genes. The reaction included 0.2 µM of each primer, 1 µl cDNA and 10 µl of GoTaq^®^ qPCR Master Mix (total 20 µl). An initial cycle was performed at 95 °C for 5 min, followed by 40 cycles (95 °C, 10 s; 60 °C, 20 s; 72 °C, 30 s). Melting curve analysis was performed to evaluate primers dimers and other artifacts. The gene expression was determined using the 2^−∆∆CT^ method [24].

### Histological evaluation

Samples from the tibiotarsal joint were fixed in 10% (v/v) buffered formalin (pH 7.4), decalcified in 10% (w/v) EDTA for 6 weeks, embedded in paraffin, sectioned (5 μm), placed on slides and stained with H&E, and sections were examined and scored by a pathologist in a blinded manner. A descriptive analysis of the samples was performed, and the following parameters were observed: cartilage surface architecture; organization and presence of chondrocytes within the lacunae; presence, intensity, and type of inflammatory infiltrate in the periarticular tissues.

### Statistical analysis

Statistical differences were determined by the Student t-test or analysis of variance (one- or two-way ANOVA), followed by the Bonferroni’s test. The results were expressed as Mean ± SEM (*n* = 8/group). For gene expression, *n* = 3/group.

## Results

### HCA reduces the levels of NO_2_^−^ and IL-1β in macrophage culture

**Fig. 1:**
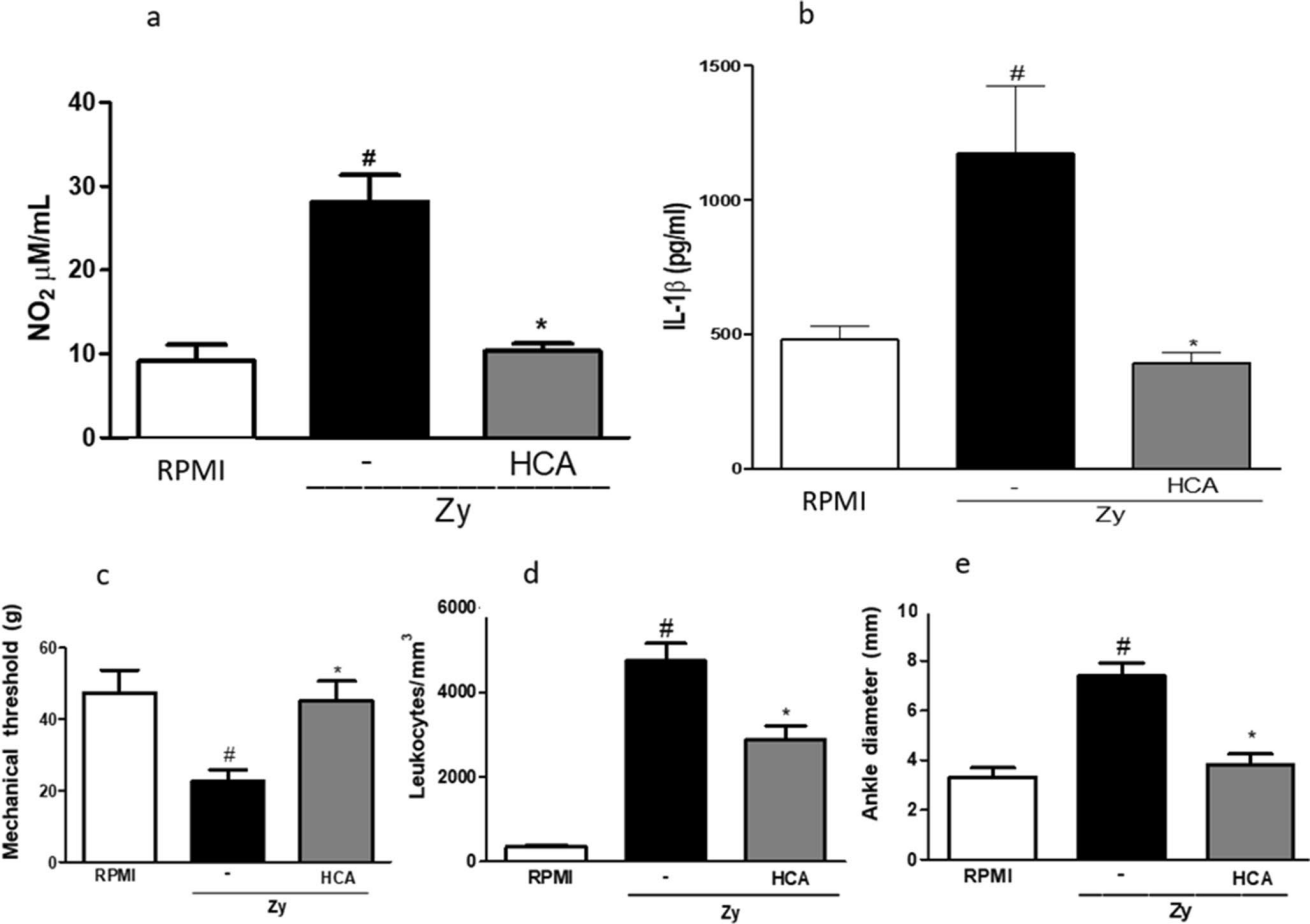
HCA inhibits the levels of NO_2_ and IL-1β in the supernatant of macrophages stimulated with zymosan and treated with HCA: re-injection of this supernatant into rat tibio-tarsal joint reduces arthritis. (**a**-**b**) Supernatants from macrophages culture were stimulated by zymosan (Zy: 10 µg/ml) before incubation with HCA (100 µg/ml; 1 h) were collected for determination of (**a**) NO_2_ and (**b**) IL-1β by enzyme-linked immunosorbent assay (ELISA). (**c**-**e**) The supernatant was re-injected (25 µl) in the tibio-tarsal joint and evaluated 6 hours later for (**c**) hypernociception (mechanical threshold), (**d**) leukocyte migration and (**e**) ankle diameter. Mean ± S.E.M. (*n*=8/group). #*p*<0.05 *vs*. RPMI,*p<0.05 *vs.*Zymosan (One-way ANOVA and Bonferroni's test)

To identify the macrophage inflammatory mediators that had been modulated in vitro by HCA, the measuring of NO_2_^−^ and IL-1β was performed. In isolated macrophages, zymosan increased the levels of NO_2_^−^ (28 ± 3.1 vs. saline: 9.1 ± 1.9 NO_2_^−^ µM/ml) and IL-1β (1172 ± 252 vs. saline: 480 ± 49.4 pg/ml), that were reversed after incubation with HCA (100 µg/ml) (NO_2_^−^: 10.3 ± 0.8 µM/ml; IL-1β: 393 ± 40.8 pg/ml) (Fig. 1a, b). These data indicate the modulator effect of HCA on the release of nitric oxide and IL-1β from articular infiltrated macrophages.

### Injection of macrophage supernatant in the rat tibio-tarsal joints inhibits the inflammatory process of zymosan-induced arthritis

After the identification of the inflammatory mediators present in the supernatant from macrophages stimulated with zymosan, it was injected into tibio-tarsal joints. The supernatant reduced the mechanical threshold (22.5 ± 4.6 vs. RPMI: 46.5 ± 6.2 g), and increased leukocyte migration (4753 ± 12 vs. RPMI: 352 ± 8 leukocytes/mm^3^) and ankle diameter (7.4 ± 0.8 vs. RPMI: 3.3 ± 1.4 mm). Supernatant of macrophages incubated for 1 h with HCA after zymosan and injected into rats increased the mechanical threshold in 2-fold (45 ± 5 g), inhibited leukocyte migration by 39% (2887 ± 12 leukocytes/mm^3^) and reversed the ankle diameter (3.8 ± 0.6 mm) (Fig. 1c, d, e). This result reinforces the involvement of IL-1 and nitric oxide on the HCA anti-inflammatory effect.

### HCA inhibits inflammatory parameters of zymosan-induced arthritis in the rat tibio-tarsal joints

**Fig. 2:**
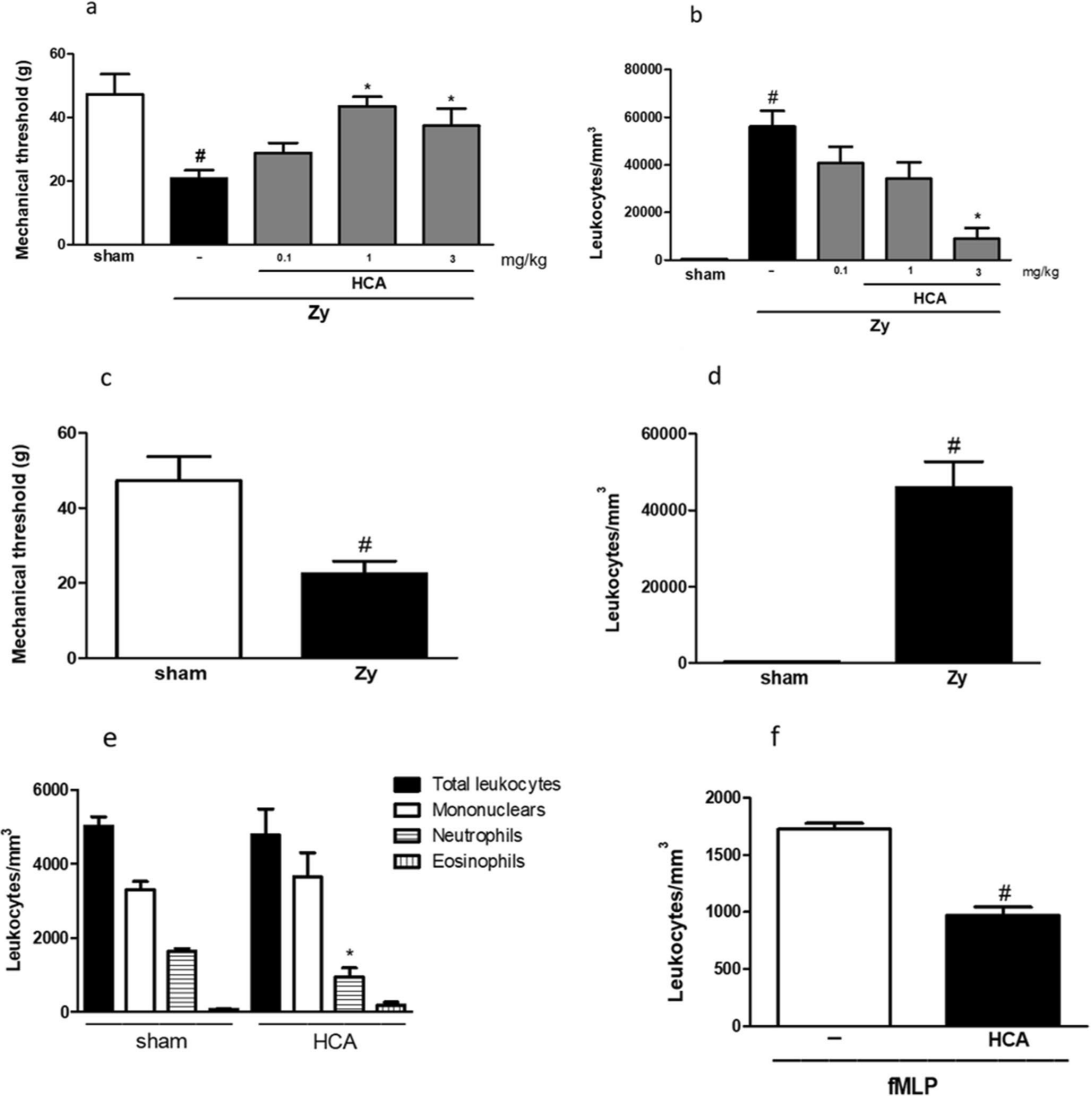
HCA causes neutropenia and reduces hypernociception and neutrophil migration by direct or indirect inflammatory stimuli.Arthritis was induced by zymosan (Zy: 500 µg/25 μl i. art.) into tibio-tarsal joint. Sham-animals received saline (25 µl; i.art.). HCA (0.1 - 3 mg/kg; i.v.) was administrated i.v. 5.5 hours after arthritis induction. (**a**, **b**) Mechanical threshold and total leukocytes at 6 h and (**c**, **d**) 5.5 h non-HCA treatment; (**e**) leukogram of non-arthritic rats 30 minutes after HCA (3 mg/kg; i.v.); (**f**) fMLP (10 nM) was injected i.art 3.5 hours before HCA (3 mg/kg; i.v.) and leukocyte migration evaluated 30 min later. Mean ± S.E.M. (*n*=8/group). ^#^*p*<0.05*vs.*sham,*p<0.05*vs.*zymosan (One-way ANOVA and Bonferroni's test)

Previously, we demonstrated that the intravenous post-treatment with HCA reduces inflammation and nociception in rat tibio-tarsal arthritis induced by direct (fMLP) or indirect (zymosan) neutrophil chemotactic agents. Thus, the HCA treatment 5.5 h after arthritis by zymosan (20.8 ± 4.5 g) reduced hypernociception at 1 mg/kg by 2-fold (43.5 ± 4.2 g) and 3 mg/kg by 1.8-fold (37.3 ± 9.7 g) evaluated 30 min later (6 h after zymosan) (Fig. 2a). At this time, HCA also inhibited leukocyte migration by 84% at 3 mg/kg (8868 ± 1568 vs. zymosan: 56030 ± 6240 leukocytes/mm^3^) (Fig. 2b). It is important to notice that either the hypernociceptive response or the increased number of leukocytes elicited by zymosan were already evidenced 5.5 h after arthritis induction (Fig. 2c, d). Besides, HCA (3 mg/kg) reduced the number of circulating neutrophils by 42% (945.4 ± 58 vs. saline: 1636 ± 32 leukocytes/mm^3^) in non-arthritic rats 30 min after injection (Fig. 2e). Moreover, HCA at the same dose reduced by 44% the leukocyte number stimulated by fMLP (893 ± 101 vs. fMLP: 1784 ± 185 leukocytes/mm^3^) (Fig. 2f).

**Fig. 3:**
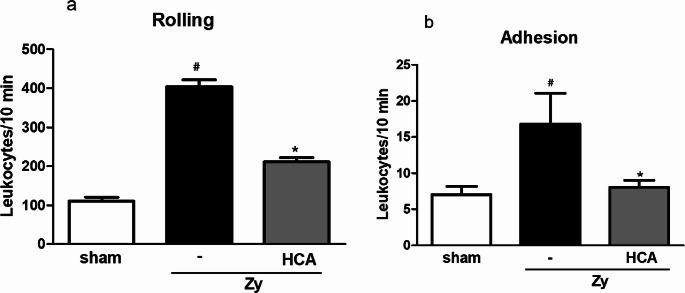
HCA inhibits rolling and adhesion of leukocytes stimulated by zymosan. Peritonits was induced by zymosan (Zy: 1 mg/100 µl; i.p.). Sham-animals received saline (100 μl i.p.). HCA (3mg/kg; i.v.) was injected 30 minutes before evaluation. (**a**) Rolling and (**b**) adhered leukocytes. Mean ± S.E.M. (n=8/group). ^#^*p*<0.05*vs.*sham,*p<0.05*vs.*zymosan (One-way ANOVA and Bonferroni's test)

The leukocyte-endothelial interactions examined by intravital microscopy showed that the leukocyte rolling and adhesion stimulated by zymosan were inhibited by HCA (3 mg/kg). The rolling was reduced by 48% (211 ± 10 vs. zymosan: 404 ± 5 leukocytes/10 min) and adhesion by 52% (8 ± 1 vs. zymosan: 16.8 ± 5 leukocytes/10 min) (Fig. 3a, b). We could conclude that HCA given by intravenous route inhibits direct and indirect neutrophil chemothactic agents, and that it may modulate local and systemic inflammatory processes.

### HCA inhibits gene expression of iNOS and IL-1 in rat tibio-tarsal joints

**Fig. 4:**
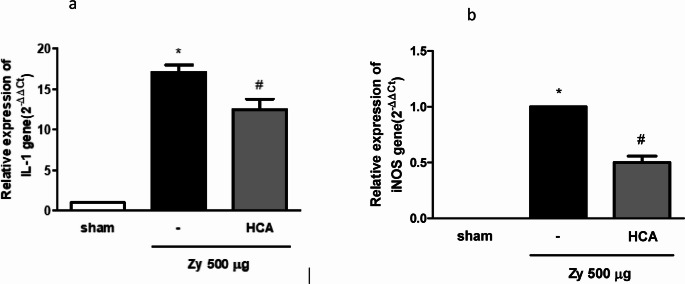
HCA inhibits gene expression of iNOS and IL-1 in rat tibio-tarsal joints. Arthritis was induced by zymosan (Zy: 500 µg/25 μl i. art.) into tibio-tarsal joint. Sham-animals received saline (25 µl; i.art.). HCA (3 mg/kg i.v.) was given 5.5 h after zymosan. qRT-PCR for (**a**) IL-1 and (**b**) iNOS. Mean ± S.E.M. (*n*=8/group). ^*^p<0.05*vs.*sham,#*p*<0.05 *vs.*zymosan.*p<0.05 *vs.*zymosan (One-way ANOVA and Bonferroni's test)

To identify the macrophage inflammatory mediators that had been modulated by HCA in vivo, NO_2_^−^ and IL-1β were measured in the periarticular tissue and fluid. Zymosan-induced arthritis increased the relative gene expression for iNOS (1 vs. saline: 0) and IL-1 (17 ± 0.9 vs. saline: 1) in the joint tissue, and HCA reduced both gene expression for iNOS (0.5 ± 0.05) and IL-1 (12.5 ± 1.3) (Fig. 4a, b). In addition, HCA reduced the levels of NO_2_^−^ by 59% (10.72 ± 1.4 NO_2_^−^ µM/ml vs. zymosan: 25.8 ± 1.4 NO_2_^−^ µM/ml) in the intra-articular fluid (data not shown).Thus, in addition to modulate systemic inflammatory process, HCA also reduce joint inflammation via attenuation of gene expression of iNOS and IL-1.

### HCA protects articular tissue

**Fig. 5:**
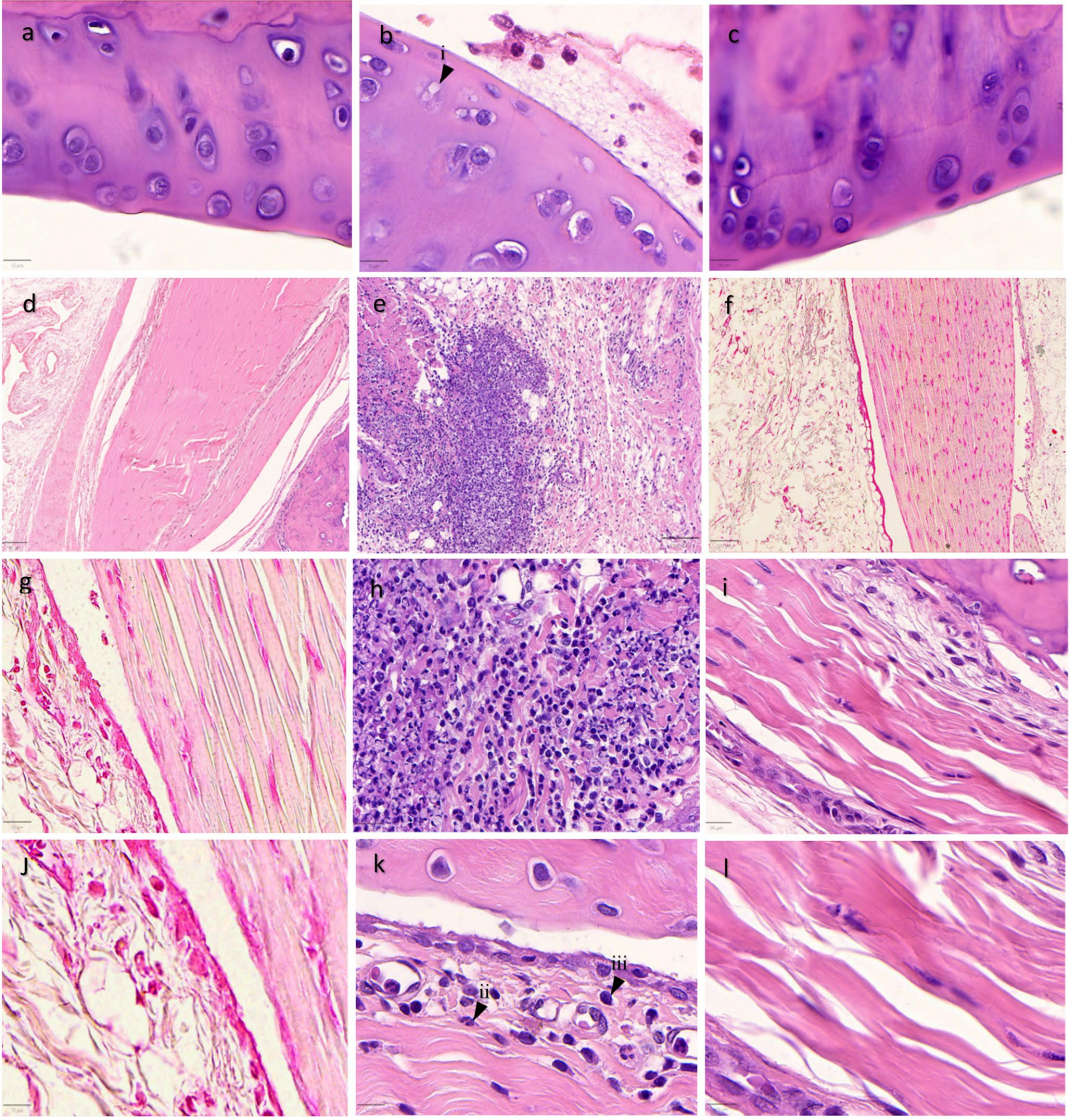
Histopathological analysis of rat joints treated with HCA.(**a**,**d**, **g**, **j**) Sham group (saline). (**b**, **e**, **h**, **k**) Zymosan group. (**c**, **f**, **i**,**l**) HCA group. (**a**-**c**) articular surface. (**d**-**l**) periarticular tissue. Zymosan (500 µg) or sterile saline was administered to tibiotarsal rat joints 2 h before treatment with HCA (3 mg/kg; *p.o*.). Areas without chondrocytes (i), polymorphonuclear (ii) mononuclear leukocytes (iii). After 6 h the tibio-tarsal joint was removed, merged into histological blocks and stained with H&E

To demonstrate the protective effect of HCA on cartilage and periarticular tissue, the histopathological analysis of tibio-tarsal joint was performed. Our data revealed that the negative control animals that received sterile saline presented normal chondrocytes and chondroblasts organized on the articular surface and periarticular tissue without inflammation (Fig. 5a, d, g, j). Tissues stimulated with zymosan showed areas of cartilage with gaps without chondrocytes, leukocyte influx to articular cavity, areas of edema and polymorphonuclear/mononuclear leukocytes influx in the periarticular tissue (Fig. 5b, e, h, k). HCA (3 mg/kg) reduced chondrocytes damage in surface and middle cartilage, and inhibited edema and leukocyte influx to the articular cavity and periarticular tissue. It was possible to demonstrate that HCA contributes to tissue protection via attenuation of inflammatory parameters in cartilage and periarticular tissues.

## Discussion

The present study demonstrated the inhibitory effect of the lectin isolated from *Hypnea cervicornis* (HCA) administered in rats by intravenous route after arthritis induction with zymosan in the tibio-tarsal joints, and the involvement of inflammatory mediators derived from macrophages.

Although the literature had already reported the antinociceptive and anti-inflammatory effects of HCA in rodents [17, 18], including in experimental arthritis model [10], it is the first time that these effects are demonstrated in post-treatment scheme, in which the animals were treated by intravenous route with HCA 5.5 h after the arthritis induction with zymosan, being the HCA effect evaluated at the peak of the inflammatory response 6 h post induction.

The HCA post-treatment inhibited the mechanical hypernociceptive response, an effect that was similar to that of morphine (opioid analgesic) and indomethacin (cyclooxygenase pathway inhibitor) at the same time of treatment [10]. The antinociceptive activity mediated by IL-1β and TNF-α inhibition had already been demonstrated for the lectin isolated from the green alga *Caulerpa cupressoides* in rat zymosan-induced arthritis [13].

In our study, zymosan evoked articular leukocyte influx, that was remarkably inhibited by HCA, showing better efficacy compared to the 2-hour post-treatment scheme, which had inhibited this parameter by 70% in the same model of zymosan arthritis [10]. This immediate reduction on the number of leukocytes may be related to cell death, since the apoptotic effect had already been demonstrated for lectins of plants [25]. It was already described that in rheumatoid arthritis (RA), activated neutrophils become resistant to apoptosis, surviving for several days compared to their normal short life of 24 h in peripheral blood [26]. However, HCA reduced not only leukocytes that had migrated to the synovial fluid, but also circulating neutrophils in the blood, suggesting a suppressor effect. Neutrophils, the most abundant cell type present in the synovial fluid of patients with RA, have high cytotoxic potential via release of proteases, inflammatory cytokines, acting as macrophages or dendritic cells in the regulation of the adaptative immune response [26].

Furthermore, the inhibitory effect of HCA on neutrophil migration was demonstrated either by direct (fMLP) or indirect (zymosan) chemotactic stimuli, which is in line with a previous study performed in the peritonitis model [17]. Zymosan stimulates toll-like receptors present in resident cells, such as macrophages, to release neutrophil chemotactic factors [27]. Thus, we may suggest the involvement of macrophages on the mechanism of HCA anti-inflammatory effect, being reinforced by the presence of mononuclear leukocytes in the periarticular tissues, detected by the histological analysis, and in vitro by the anti-inflammatory effect of HCA in isolated macrophages.

It had been described that the intraperitoneal injection of zymosan into rodents stimulates acute peritonitis, characterized by massive neutrophil migration [28, 29]. Accordingly, the HCA anti-inflammatory effect was confirmed in the peritonitis model. Although the effect of lectins isolated from higher plants had already been described on the steps of leukocyte migration [30–32], the activity of an alga lectin is still unknown.

As seen by intravital microscopy, HCA reduced the number of leukocytes rolling and adhesion on the endothelium of mesenteric vessels. This is highly suggestive of a relevant regulation of endothelial adhesion receptors, such as selectins, responsible for slow rolling, and integrins and immunoglobulins involved in cell adhesion.

Macrophages promote the release of TNF-α, IL-1β, thrombin and histamine, which increases the binding affinity of endothelial adhesion molecules [33]. Moreover, N-acetylgalactosamine associated with HCA partially reversed the HCA effect on leukocyte migration [10], probably by a competitive interaction at the selectin binding sites on leukocyte and/or endothelial cells membranes, as previously suggested [17, 18]. In addition, we can also consider the inhibitory effect of HCA on gene expression of important mediators of the leukocyte migration process, such as iNOS and IL1.

The cytokine IL-1 is released mainly from macrophages, monocytes and platelets [5], playing central role in the pathogenesis of RA, increasing bone resorption, cartilage degradation, reduction of collagen, and activation of metalloproteinases and endothelial cell activation [6]. Intravenous administration of HCA in rats before induction of paw edema by carrageenan [17] or 2 h after zymosan-induced arthritis [10] did not inhibit the production of IL-1. In our study HCA, administered 5.5 h after arthritis, reduced the relative gene expression for IL-1 in the intra-articular tissue. Thus, we hypothesized that there is a progressive reduction in the lectin activity due to its in vivo degradation along the evaluation period. Moreover, in vitro, HCA reversed the levels of IL-1β in the macrophage supernatant, suggesting inhibition on cytokine release by macrophages.

NO produced by iNOS is involved in the maintenance of the inflammatory response, mediating vasodilation, increased vascular permeability, nociception and leukocyte chemotaxis [34]. There is considerable amount of evidence pointing to the role of NO in the experimental arthritis induced by zymosan [10, 33–35]. High levels of NO have been associated with the development of arthritis [7], while the administration of NO synthase (NOS) inhibitors reverses inflammatory alterations in rat models of zymosan-induced arthritis [35, 36].

In a previous study, HCA reduced inflammatory nociception by mechanisms involving NO in mice classical models [18]. It is possible that the attenuation of iNOs results in decreased NO levels in the joint tissue. In fact, our study demonstrated that HCA reduced the iNOS gene expression in periarticular tissue and nitrite concentrations in the macrophage supernatant. Accordingly, the 2-hour post-treatment with HCA after arthritis induction by zymosan also decreased the gene expression of iNOS [10]. This could be explained by the fact that NO is a mediator released in both early and late phases of inflammation [37].

It is known that high concentrations of NO generate peroxynitrite, a highly oxidizing radical [38], that reduces cellularity in cartilage tissue and alters cartilaginous tissue [36, 39]. As observed in the histopathological analysis, HCA preserved chondrocytes, attenuated tissue edema and diminished the amount of polymorphonuclear and mononuclear leukocytes in the periarticular tissue.

Moreover, the intra-articular administration of zymosan enhances oxidative stress markers in rodents on different arthritis models [40, 41]. Since HCA reduced nitrite levels in the intra-articular fluid in vivo and in the macrophages supernatant in vitro, it seems to play an important role as antioxidant in the zymosan-induced arthritis. HCA also reduced the levels of the pro-oxidant enzyme myeloperoxidase in paw tissues of rats challenged with carrageenan [17].

Zymosan is also a Toll-like receptor-TLR agonist, whose activation stimulates signaling pathways that induce expression of iNOS and pro-inflammatory cytokines such as IL-1β via NF-κB in several cells, including macrophages [27]. To confirm whether HCA inhibits inflammatory parameters stimulated by zymosan via reduction of inflammatory macrophage-releasing mediators, macrophages were stimulated with zymosan and incubated with HCA. The resulting supernatant was injected into the tibio-tarsal joints of rats, being the inflammatory process evaluated 6 h after injection. This supernatant reduced hypernociception, leukocyte migration and articular edema, corroborating the in vitro tests and reinforcing the important role of macrophage as target for HCA to exert the anti-inflammatory effect. Since either iNOS inhibitors and NO donors decrease articular incapacitation and hypernociception in rat models of zymosan-induced arthritis without affecting edema [35, 36], along with the inhibitory effect of HCA on iNOS and IL-1, we may suggest that both mediators are implied on hypernociception and leukocyte migration.

Zymosan is known to enhance the production of IL-1β from macrophages and neutrophils, and IL-1β is also known to enhance expression of lectin-like ox-LDL receptor-1 (LOX-1), playing multiple roles in arthritis. A previous report suggested that the LOX-1-mediated proinflammatory mechanism may be involved in the pathogenesis of murine arthritis induced by zymosan [42]. It is known that the blockage of LOX-1 by lectin-like molecules offers potential therapeutic approach to reduce inflammation, considering the immunomodulator effect of HCA on macrophage-derived cytokine levels and leukocytes migration. Thus, it is possible to be hypothesized the modulator role of HCA on immune cells and chondrocytes, due to its capacity of binding to glycoconjugates, conferring tissue protection in the tibio-tarsal arthritis induced by zymosan.

Although our data may suggest HCA as potential therapeutic alternative for arthritis, the translation of these findings to humans deserves several concerns, since its parenteral administration ensures bioactivity, but requires careful monitoring for immunogenicity. Thus, HCA could be used as an adjunctive therapy, in association with the conventional disease-modifying antirheumatic drugs to reduce dosing requirements and improve inflammatory control. Moreover, further studies are needed to determine effectiveness and safety in humans.

In conclusion, HCA exerts anti-inflammatory effect in post-treatment scheme at the peak of inflammation induced by zymosan in tibio-tarsal joint of rats, involving NO and IL-1 released by resident macrophages.

## Supplementary Information

Below is the link to the electronic supplementary material.

Supplementary Material 1 (DOCX 4.40 MB)

## Data Availability

No datasets were generated or analysed during the current study.
