# Supplementary material for: Macrophage modulation accounts for the anti-inflammatory effect of Hypnea cervicornis agglutinin in rat arthritis induced by zymosan
Source: Glycoconj J. 2026 Jun 22;43(1):23. doi: 10.1007/s10719-026-10221-5 (PMC13287125; doi:10.1007/s10719-026-10221-5)
Supplement: Supplementary file 1 — Supplementary Material 1 (DOCX 4.40 MB) [file 10719_2026_10221_MOESM1_ESM.docx]

Below are descriptions of the total RNA samples extracted from animals with Zymosan-induced arthritis and lectin treatment and applied to 1.2% agarose gel.

Saline group: 05 animals (1 animal died)

Zymosan group: 05 animals

Treatment group 1: 05 animals

Treatment group 2: 05 animals

Total RNA samples were extracted in duplicate.

In gel A, only one of the duplicates of the total RNA samples from the saline group (lanes 1 to 4) and the zymosan group (lanes 6 to 9) were applied to the agarose gel (Figure in Gel A).


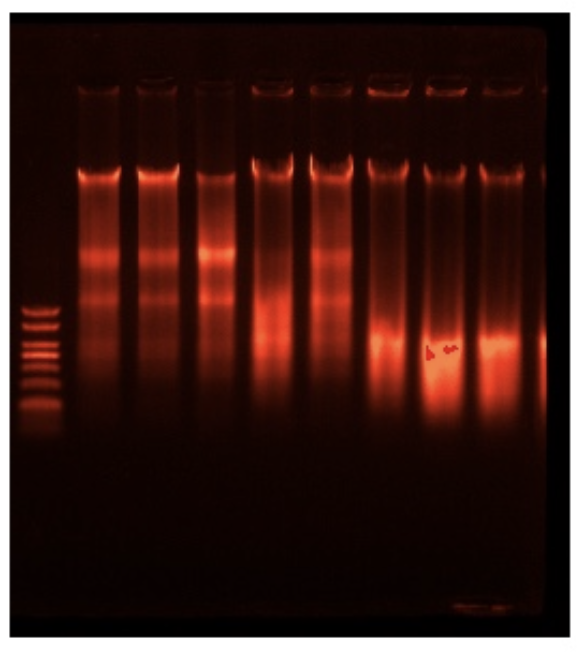


1 2 3 4 5 6 7 8 9

**Figure Gel A**

Lanes:
1 – Molecular RNA ladder

2- Animal 1 saline group

3- Animal 2 saline group

4- Animal 3 saline group

5- Animal 4 saline group

6- Animal 1 zymosan group

7- Animal 2 zymosan group

8- Animal 3 zymosan group

9- Animal 4 zymosan group

In Gel B, samples from the zymosan group were applied to lanes 2 and 3 (reapplication of animal samples 3 and 4 from Gel A), duplicates of total RNA samples from the 5 animals in treatment group 1 (lanes 4 to 13) were also applied and duplicates of total RNA samples from animals 1 and 2 of treatment group 2 (lanes 14 to 17).


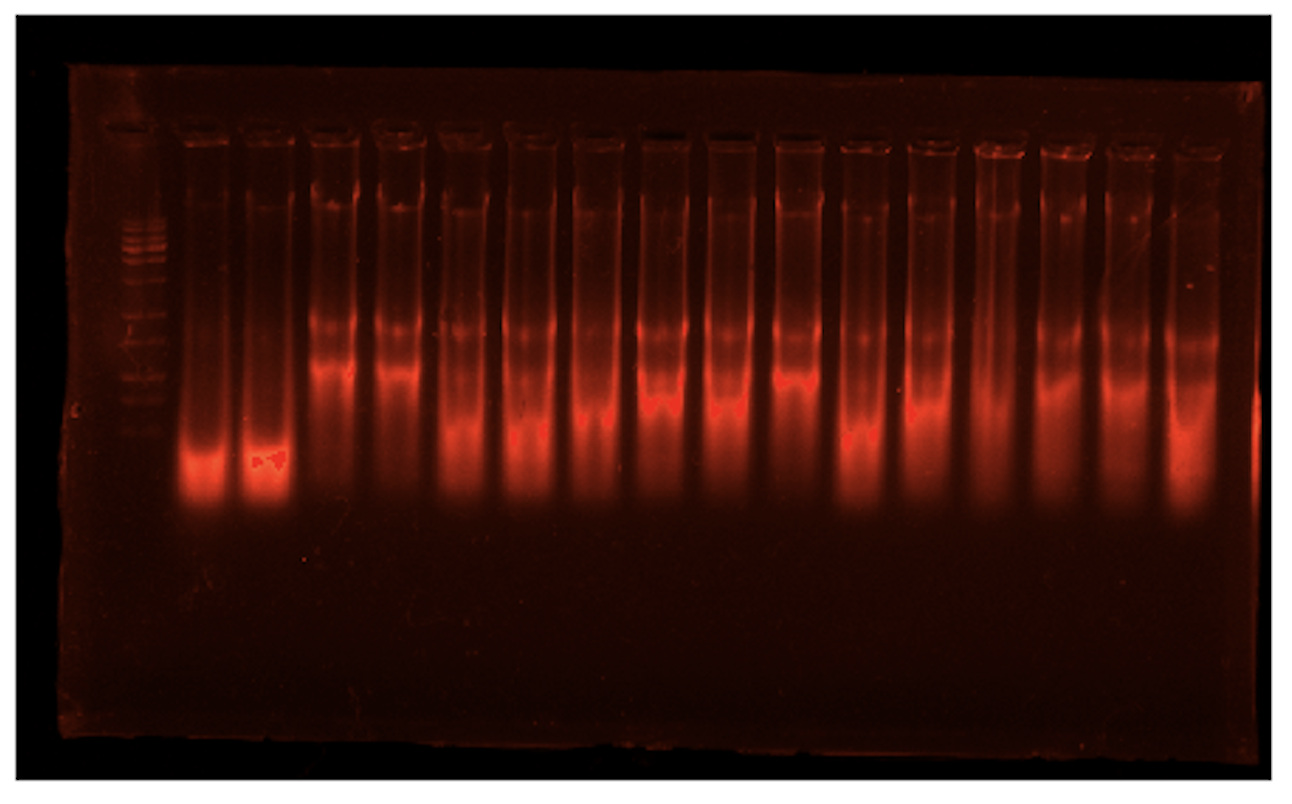


1 2 3 4 5 6 7 8 9 10 11 12 13 14 15 16 17

**Figure Gel B**

Lanes:
1 – Molecular RNA ladder

2- Animal 3 Zimosan group (sample reapplication)

3- Animal 4 Zimosan group (sample reapplication)

4- Animal 1 treatment group 1

5- Animal 1 treatment group 1

6- Animal 2 treatment group 1

7- Animal 2 treatment group 1

8- Animal 3 treatment group 1

9- Animal 3 treatment group 1

10- Animal 4 treatment group 1

11- Animal 4 treatment group 1

12- Animal 5 treatment group 1

13- Animal 5 treatment group 1

14- Animal 1 treatment group 2

15- Animal 1 treatment group 2

16- Animal 2 treatment group 2

17- Animal 2 treatment group 2

**GEL C:**

Duplicates of Total RNA samples from animals 3 to 5 of treatment group 2 (lanes 2 to 7) were applied to Gel C. Lane 8 represent excess sample applied into lane 7.


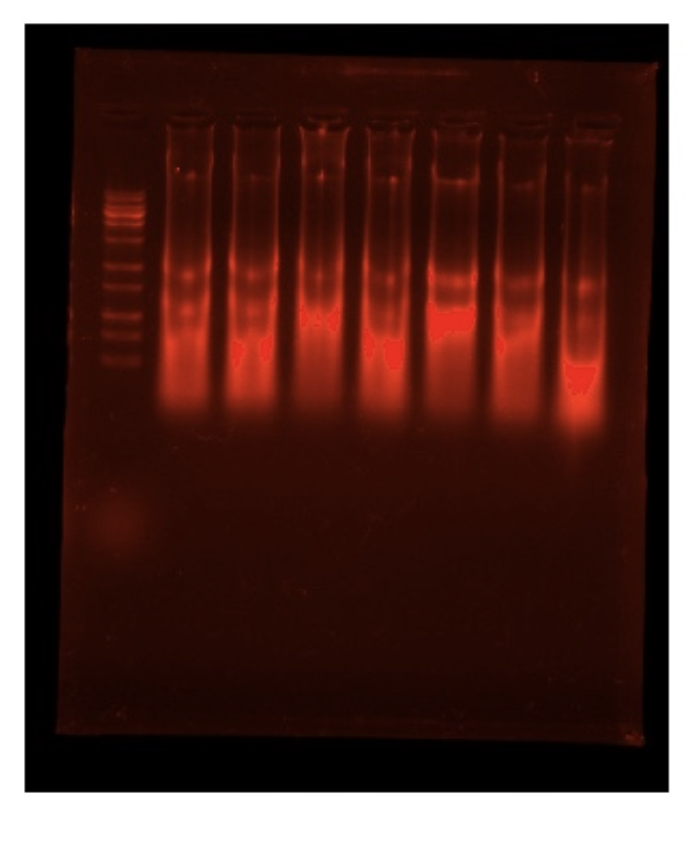


1 2 3 4 5 6 7 8

**Figure Gel C**

Lanes:
1 – Molecular RNA ladder

2- Animal 3 treatment group 2

3- Animal 3 treatment group 2

4- Animal 4 treatment group 2

5- Animal 4 treatment group 2

6- Animal 5 treatment group 2

7- Animal 5 treatment group 2

8- Animal 5 treatment group 2
